# It’s Out of My Hands! Grasping Capacity May Not Influence Perceived Object Size

**DOI:** 10.1037/xhp0000331

**Published:** 2017-02-13

**Authors:** Elizabeth S. Collier, Rebecca Lawson

**Affiliations:** 1 Department of Experimental Psychology, University of Liverpool

**Keywords:** action, action capacity, perception, touch, vision

## Abstract

Linkenauger, Witt, and Proffitt (2011) found that the perceived size of graspable objects was scaled by perceived grasping capacity. However, it is possible that this effect occurred because object size was estimated on the same trial as grasping capacity. This may have led to a conflation of estimates of perceived action capacity and spatial properties. In 5 experiments, we tested Linkenauger et al.’s claim that right-handed observers overestimate the grasping capacity of their right hand relative to their left hand, and that this, in turn, leads them to underestimate the size of objects to-be-grasped in their right hand relative to their left hand. We replicated the finding that right handers overestimate the size and grasping capacity of their right hand relative to their left hand. However, when estimates of object size and grasping capacity were made in separate tasks, objects grasped in the right hand were not underestimated relative to those grasped in the left hand. Further, when grasping capacity was physically restricted, observers appropriately recalibrated their perception of their maximum grasp but estimates of object size were unaffected. Our results suggest that changes in action capacity may not influence perceived object size if sources of conflation are controlled for.

## Public Significance Statement

The action-specific account of perception suggests that an observer’s capacity for action scales how the environment appears to them and, specifically, how they perceive its spatial properties. However, contrary to the predictions of this account, the results of the present studies suggest that perceived object size is not influenced by either actual or perceived grasping capacity. First, although right handers perceived their right hand to be both larger and to have a greater grasping capacity than their left hand, size estimates for an object were not influenced by which hand was used to grasp that object. Second, in a stronger manipulation, we reduced both the actual and the perceived grasping capacity of one hand by taping its fingers together. Despite this causing a substantial reduction in action capacity, it did not influence estimates of object size. These results show that action capacity and spatial properties can be perceived independently.

Action capacity refers to an observer’s ability to perform a given action. Physical changes to the body can alter both actual and perceived action capacity. The action-specific account of perception claims that observers perceive features of the environment as scaled according to their abilities (Proffitt, 2006a, 2006b, 2013; Proffitt & Linkenauger, 2013; Witt, 2011a). Spatial perception has been shown to scale according to energetic expenditure and effort (Bhalla & Proffitt, 1999; Proffitt, Bhalla, Gossweiler, & Midgett, 1995; Proffitt, Stefanucci, Banton, & Epstein, 2003; Witt, Proffitt, & Epstein, 2004) and performance variability (Witt, Linkenauger, Bakdash, & Proffitt, 2008; Witt & Dorsch, 2009). For example, proponents of the action-specific account have claimed that hills appear steeper when observers wear a heavy backpack or are fatigued (Bhalla & Proffitt, 1999), that putting holes and softballs appear larger (Witt et al., 2008; Witt & Proffitt, 2005) and that tennis balls appear to move slower (Witt & Sugovic, 2010) to more skilled players of the relevant sport. According to the action-specific account, perception is scaled in these ways to guide effective actions (Bhalla & Proffitt, 1999; Proffitt & Linkenauger, 2013). For example, an observer wearing a heavy backpack will find it harder to walk up a hill and so the visual slant of the hill appears steeper to them in order to deter them from attempting the ascent.

It has also been reported that perception may be influenced by action capabilities pertaining to the functional morphology of the body (Linkenauger, Ramenzoni, & Proffitt, 2010; Linkenauger, Witt, & Proffitt, 2011). For example, observers estimate an object to be nearer when they hold a tool that increases their maximum reach (Witt, Proffitt, & Epstein, 2005) and apertures are estimated as narrower if observers hold a horizontal rod that is wider than their body (Stefanucci & Geuss, 2009). Further evidence for the action-specific account comes from the claim that right-handed participants underestimate the size of objects they intend to grasp with their right hand relative to objects they intend to grasp with their left hand (Linkenauger, Witt, & Proffitt, 2011; see also Linkenauger et al., 2010). Linkenauger and colleagues claim that this is because right-handed observers perceive that their right hand is larger than their left hand and therefore that it can grasp larger objects (Gentilucci, Daprati, & Gangitano, 1998; Linkenauger, Witt, Bakdash, Stefanucci, & Proffitt, 2009).

Action-specific scaling effects challenge modular theories of vision, as they suggest that perception can be influenced by cognitive factors. Modular theories of perception claim that perception is *cognitively impenetrable,* that is perception is not affected by top-down, cognitive influences (for discussions see Firestone, 2013; Firestone & Scholl, 2014, 2015; Proffitt, 2013). However, Sugovic, Turk, and Witt (2016) recently pointed out that if action-specific scaling effects are driven by real, physical body morphology (e.g., actual weight) rather than beliefs or thoughts about the body, then these effects are, in fact, compatible with the idea of cognitive impenetrability. This is because information about the physical abilities of the body—rather than conscious beliefs or thoughts—is influencing perception, possibly through multimodal processes. This information need not be specified in the visual array, but instead may, for example, be provided by other modalities or physiological cues. As Firestone and Scholl (2015, p.11) suggest, for multisensory integration, “such results are consistent with the entire process being contained within perception itself, rather than being an effect of more central cognitive processes on perception.”

Interestingly, Sugovic et al. (2016) found that only actual weight, and not beliefs or perceptions about body mass, predicted action-specific scaling effects—in this case, distances were estimated as greater by heavier observers. This finding, namely that only actual, and not perceived, body morphology influenced spatial estimates contrasts to Linkenauger et al.’s (2011) finding that it was people’s perceptions of their grasping capacity that scaled their estimates of object size, whereas their actual grasping capacity did not differ between the right and left hands.

One concern with the action-specific account is that the reported scaling effects may not reflect changes to perceived size in the strongest sense. Instead, participants’ size estimates may reflect their perception of their ability to act on an object as opposed to being based on the object’s spatial properties alone. A conflation of perceived action capacity and spatial perception is more likely to occur when spatial estimates are made in a context which encourages participants to consider nonvisual factors, possibly including their action capacity (Firestone, 2013; Woods, Philbeck, & Danoff, 2009).

Woods et al. (2009) demonstrated this possibility experimentally. Participants threw either a light or a heavy ball to a target three times, and then verbally estimated the distance to the target. Participants in three different groups were asked to base their distance judgments on objective distance (how far away the target really was), apparent distance (how far away the target visually appeared to be), or nonvisual factors (how far away they ‘felt’ the target was). Action-specific scaling was considered to have occurred if the distance to the target appeared greater to those who threw the heavy ball, since more effort is needed to throw a heavy than a light ball (see Proffitt et al., 2003; Witt et al., 2004). Woods et al. (2009) found that action-specific scaling occurred only for participants judging how far they ‘felt’ the target was. Only here were participants encouraged to consider nonvisual factors, which may have included their throwing ability. This result suggests that the scaling effect obtained by Woods et al. arose from a difference in how easily participants could throw the ball to the target, and did not actually reflect a change in what they perceived visually.

We investigated this issue by reexamining the results of a study conducted by Linkenauger et al. (2011) where right-handed participants were presented with blocks of varying size. On each trial in Linkenauger et al. (2011), participants were first asked whether they thought they could grasp the block with either their left or right hand. They then visually matched the width of the block on a screen by moving two circles apart. Participants estimated the grasping ability of their dominant right hand as greater than that of their left hand. Critically, participants also underestimated the size of blocks they had imagined grasping with their right hand to a greater extent than blocks they had imagined grasping with their left hand. These findings were taken to demonstrate a scaling of perceived object size according to perceived action capacity. However, on each trial participants were explicitly asked whether they would be able to grasp the block with their thumb on one side and any finger on the other side immediately before they estimated the block’s width. It is therefore possible that their estimates of object size were influenced by whether the block seemed graspable, rather than its objective size alone.

Linkenauger et al. (2011) asked participants to imagine grasping the blocks because it has sometimes been argued that observers must intend to act in order for action-specific effects to be found (Witt et al., 2005; Witt & Proffitt, 2008). However, action-specific scaling effects have been found when participants performed the relevant action without being asked to consider doing it. For example, in Bhalla and Proffitt’s (1999) studies with backpacks no attention was drawn to action when slopes were estimated. In a further example, Witt and Dorsch (2009) asked participants to attempt 10 kicks to a set of field goal posts and then to visually match the height of the goal posts. When they estimated height they were not encouraged to consider their previous kicks and they did not kick the ball again after making their estimate and so they were not anticipating further action.

It could be argued that this example reflects a different kind of perceptual scaling to that measured by Linkenauger et al. (2011). Specifically, in Witt and Dorsch (2009), spatial properties were scaled by variability in performance, whereas in Linkenauger et al. (2011) spatial properties were scaled by functional morphology (for a discussion of this issue, see Proffitt & Linkenauger, 2013). Nevertheless, the results of Witt and Dorsch (2009) suggest that action does not need to be consciously considered in order for action-specific effects to occur. Furthermore, if people know they have to perform a given action they must intend to act even if they are not consciously considering that action. Thus if intention is sufficient to influence perception, then performing the relevant action should carry the same biases as imagining doing so. In addition, actually performing an action creates a more ecologically valid context in which to test the claims of the action-specific account. Thus in our studies, participants actually grasped a block on each trial, rather than only imagining grasping a block, as in Linkenauger et al. (2011).

We conducted five experiments investigating whether spatial perception is scaled by perceived action capacity. We focused on the claim made by Linkenauger et al. (2011) that right-handed observers estimate the size of objects they intend to grasp in their right hand as smaller than objects they intend to grasp in their left hand because they perceive the grasping capacity of their right hand as greater than that of their left hand. However, we avoided conflation effects by dissociating estimates of action capacity from estimates of object size. In Experiments 2 through 4 we did this by asking our participants to estimate their grasping capacity in a separate task which was completed only after they had made all of their estimates of object size. In the final experiment (5) we did this by deceiving participants by giving them a cover story that the action capacity and object size estimate tasks were unrelated and were part of two separate studies. Thus we investigated whether a difference in either perceived or actual grasping capacity predicted a difference in perceived object size when potential sources of conflation were avoided. If perception is cognitively penetrable, and so if it is influenced by perceived action capacity, then we should replicate Linkenauger et al.’s (2011) results when participants actually perform the relevant action, in this case grasping. In contrast, failure to replicate these effects when the action is performed and conflation effects are controlled for would support the claim that perception is cognitively impenetrable (Firestone, 2013; Firestone & Scholl, 2015).

## Experiment 1

Linkenauger et al. (2009) reported that right-handed participants perceived that they could grasp larger objects in their right hand than in their left hand. This may reflect an asymmetry in the perceived size of the hands, such that the right hand is perceived as larger than the left, since right-handers rely on their right hand more. A similar asymmetry has been reported for arm length, where the right arm is perceived by right-handed individuals to be longer than the left arm (Linkenauger et al., 2009; Morgado, Gentaz, Guinet, Osiurak, & Palluel-Germain, 2013). To test the robustness of the claim that right handers perceive their right hand as bigger than their left hand, in Experiment 1 we asked participants which of their hands was bigger.

### Method

#### Participants

An opportunity sample of 50 participants who self-reported as right-handed (25 females, mean age = 23.3 years) was recruited for this experiment. Thirty-nine participants were approached in person on the University of Liverpool campus and 11 were questioned online via Skype.

#### Stimuli and procedure

The experimenter recorded the participant’s age, gender, and handedness then asked “Is your right hand smaller, larger, or about the same size as your left hand?” If they responded that they believed their hands were about the same size, they were asked the follow-up question, “If I forced you to choose, which is bigger, your right or left hand?” Participants often looked at their hands before they made their judgment.

### Results and Discussion

A chi-square test of goodness-of-fit for the participants who responded right (*n* = 25) and left (*n* = 10) to the first question showed a significant difference, χ^2^(1, *N* = 35) = 6.4, *p* < .001. We repeated this analysis including participants who responded right (*n* = 14) or left (*n* = 1) to the second question and again found a significant difference, χ^2^(1, *N* = 50) = 15.7, *p* < .001. Thus right handers were more likely to say their right hand was larger than their left hand than vice versa. This supports the claim made by Linkenauger et al. (2009) that most right-handed observers perceive their right hand as larger than their left hand.

## Experiment 2

Having confirmed that right handers perceive their right hand as larger than their left hand in Experiment 1, in Experiment 2 we went on investigate whether this effect would lead to the size of objects grasped by the right hand being underestimated relative to those grasped by the left hand (Linkenauger et al., 2011). The action-specific account claims that this should occur because perceived action capacity alters the perceived size of action-relevant objects. As explained by Linkenauger et al. (2011, p. 1436): “Because the right hand appears larger and is deemed to be able to grasp larger objects (Linkenauger, Witt, Bakdash, et al., 2009), the same object measures as smaller on the right hand’s larger ruler, and therefore, appears smaller than when it is placed on the left hand.”

In addition to testing for a preexisting bias to overestimate the grasping ability of the right hand, in Experiment 2 we tried to manipulate perceived grasping ability in a second way, by using a priming task. Franchak and Adolph (2014) showed that changes to the body are not sufficient to change perceived action capacity but that perceived action capacity may be recalibrated through acting. They demonstrated this by comparing pregnant and nonpregnant participants’ estimates of their ability to walk through apertures of different widths. Pregnant participants accurately updated their estimates of the narrowest aperture that they could squeeze through as their body size increased throughout their pregnancy. In contrast, nonpregnant participants who were fitted with a pregnancy prosthesis that immediately increased their girth were initially poor at estimating the narrowest aperture they could fit through. However, after physically attempting the task their estimates became accurate. Thus perceived action capacity can be quickly recalibrated through acting (see also Franchak, van der Zalm, & Adolph, 2010; Ishak, Adolph, & Lin, 2008).

We aimed to take advantage of this rapid recalibration in Experiment 2 by priming participants to feel that one of their hands had a greater grasping capacity than the other hand prior to estimating the size of objects. One group was primed to feel their right hand was able to grasp larger objects. Here, any preexisting bias to overestimate the grasping ability of their right hand should have been enhanced. If this bias influences estimates of object size, then any scaling effects should also have been enhanced. The other group were primed to feel their left hand could grasp larger objects. Here, any priming effect should have countered a preexisting bias to overestimate the grasping capacity of their right hand. This, in turn, should reduce or even reverse any scaling effects when estimating object size.

Finally, in Experiment 2 we also tested whether perceived grasping capacity would influence perceived object size if objects were presented haptically as well as visually. Both vision and active touch (haptics) process spatial information (Collier & Lawson, 2016; Lawson, 2009; Lawson, Ajvani, & Cecchetto, in press). Active exploration of the environment is critical to learning about the action capacity of the hands, and Gori and colleagues have shown that haptic information calibrates visual estimates of object size in young children (Gori, Del Viva, Sandini, & Burr, 2008; Gori, Sciutti, Burr, & Sandini, 2011). Some evidence suggests that the direction of perceptual scaling effects may reverse from vision to touch. For example, Taylor-Clarke, Jacobsen, and Haggard (2004) found that magnifying the forearm led participants to estimate visual stimuli presented on the forearm as smaller. However, they found that when the forearm was again visually magnified but unseen stimuli passively touched the skin, objects were estimated as larger. Similarly, using the rubber hand illusion, Bruno and Bertamini (2010) found that when participants embodied a large hand, they estimated disks that they grasped in that hand as larger than when they embodied a small hand. This research suggests that differences in the perceived size of the relevant body part can elicit opposite perceptual scaling effects for vision and touch. Applying this size-scaling logic to the current studies, if right-handed participants perceive their right hand to be larger than their left hand then objects they grasp in the absence of vision may be perceived as larger in the right hand, which is the opposite prediction to that of the action-specific account.

### Method

#### Participants

Thirty right-handed undergraduate students from the University of Liverpool were recruited for this study (21 females, mean age = 20.6 years, mean Edinburgh Handedness Inventory score = 86, range = 25–100). Participants were rewarded with course credit for their participation.

#### Stimuli, apparatus, and procedure

There were four phases to this experiment. In summary, first participants were implicitly primed to perceive one of their hands as having a greater grasping capacity than the other (priming task). Second, they completed a haptic-to-vision size estimation task (HV task) where they used a visual matching response to estimate the size of haptically presented stimuli. Third, they repeated the HV task but this time the stimuli were presented visually (VV task). Finally, we measured the largest object that participants could grasp with each hand (grasping capacity task). These four phases are described in more detail below.

**Figure 1 fig1:**
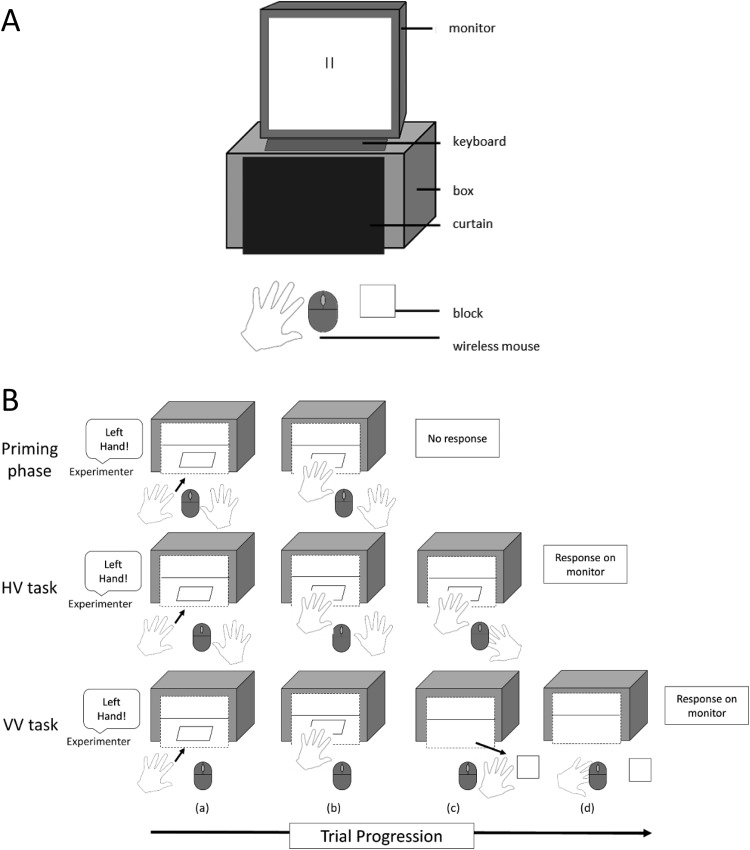
(A) Diagram of the set-up of Experiment 2, showing a left hand trial in the VV task. The participant has moved the block from behind the curtain and placed it on the table in front of them, to the right of the mouse. They would then use their left hand to scroll the mouse wheel to respond. (B) Diagram showing the procedure during left hand trials in Experiment 2 in the priming phase (top), the HV task (middle) and the VV task (bottom). The same procedure was used in Experiment 3 (except that the HV task was omitted), Experiment 4 (except that the priming phase was omitted). In Experiment 5 both the priming phase and HV task were omitted and changes were made to the VV task. In 1B, unlike in 1A, the curtain is drawn as transparent to show the block placed behind the curtain. In fact, though, participants could not see the block while it was behind the curtain (in (a) and (b) and also in (c) for the HV task). On left hand trials in the HV task, the left hand was used to feel the block, while the right hand was used to respond using the mouse. In the VV task, the right hand was not used and was kept out of sight, while the left hand was used to move the block from behind the curtain, to place it to the right of the mouse and then to respond using the mouse.

Participants were first told that the experiment would test their ability to estimate the size of blocks. The stimuli were 21 foamboard square blocks (0.5 cm deep) with sides varying in length from 4 to 24 cm in 1-cm increments. A box (40 × 10 × 32 cm) was placed on top of a table at which participants were seated. The open end of the box facing the participant was covered by a curtain, see Figure 1A. Stimuli were presented inside the box in the priming phase and in the HV and VV tasks.[Fig-anchor fig1]

The purpose of the priming phase was to induce the feeling that one hand could grasp larger objects than the other by giving a smaller set of objects to that hand. We reasoned that if participants were able to grasp more blocks with one hand than the other, they could be led to perceive that hand as having a greater grasping capacity if they assumed that the same set of stimuli were being given to both hands. Any difference in graspability between the two hands might subsequently lead to objects seeming smaller when seen near to that hand. Participants were assigned to either the LHFeelsSmallerObjects or the RHFeelsSmallerObjects group (*n* = 15 per group) and were given a series of stimuli to try to pick up.

As a cover story for the priming phase, participants were told that before starting to estimate object sizes, they would do a practice phase in which they would feel objects from across the range of available sizes without making a response. In this phase participants reached behind the curtain with their left or right hand and attempted to grasp and pick up the presented block. The experimenter told the participant which hand they should use on each trial. Participants were told to always attempt to first grasp the square block with their thumb on one side and any other finger on the opposite side. They were also told that if the block was too big to grasp in this way, they should then move their hand across the block to feel its width. There were two sets of 13 stimuli, the small set (sizes = 4, 5, 6, 7, 8, 10, 12, 14, 16, 17, 18, 19, and 20 cm) and the large set (each member of which was 4 cm larger than its corresponding item in the small set, so its sizes = 8, 9, 10, 11, 12, 14, 16, 18, 20, 21, 22, 23, and 24 cm). For the LHFeelsSmallerObjects group, on each trial one block from the small set was presented to the left hand and then the corresponding (4 cm larger) block from the large set was presented to the right hand, and vice versa for the RHFeelsSmallerObjects group. Each pair of blocks was presented twice, giving 26 trials in total. Trial order was randomized for each participant, and the hand given the small set (so being primed to have a greater grasping capacity) acted first on every trial.

The HV then VV size estimation tasks followed immediately after the priming phase (see Figure 1B). At the beginning of each trial, the experimenter told the participant which hand they should use to grasp the block. In the HV task, participants put their hand through the curtain to feel the block, as in the priming phase. In the VV task, participants reached through the curtain to pick up the block and placed it on the table in front of the curtain so that they could see it. In both tasks, participants always attempted the specified grasp (with the thumb on one side and any other finger on the opposite side) first. However, if the block was too big to pick up in this way then they were told to move their hand across the block to feel its width (for the HV task) or they were told to use a different grasp to pick up the block (for the VV task). Thus in both tasks participants attempted to grasp the block in a specific way on every trial prior to estimating its size.

For the HV and VV tasks, size estimates were made on a computer monitor, which was placed on top of the box. Two 2-cm tall, 0.5-cm wide, vertical black lines, which were initially 1.75 cm apart, were displayed on the screen, see Figure 1A. The participant moved the lines closer or further apart by scrolling the wheel of a wireless mouse. The mouse was fixed to the table in front of the participant, in line with their body midline. To estimate the width of each block, participants adjusted the horizontal distance between the lines until they believed it matched the width of the block they were either feeling (HV task) or seeing (VV task; here, the block was offset from the two lines, see Figure 1A. This ensured that participants could not simply line up the edges). Participants pressed the space key on a keyboard placed on top of the box in front of the monitor when they were satisfied with their response.

In the HV task, participants felt the block with one hand and used their other hand to scroll the mouse wheel. In the VV task, they used the same hand they picked the block up with to use the mouse and they were told to keep their other hand out of sight (as a cover story, participants were told that this was to ensure that their other hand did not get in the way, and so that they could clearly see the block). This ensured that the hand they had just acted with was more likely to then be used as a perceptual ruler as it was the only hand visible. Participants estimated the size of each of the blocks once for each hand in each task, thus completing 42 trials (2 hands × 21 blocks) in each task, with trial order randomized within each task.

After completing the VV task we measured the largest block that participants were able to successfully grasp with each hand (grasping capacity task). Participants attempted to grasp blocks, starting at 14 cm wide, in increasing size until the largest block they could grasp was found. Only actual, not perceived, maximum grasp was measured in Experiment 2. Participants then completed the 4-item short Edinburgh Handedness Inventory. Finally, to check for demand characteristics, participants were asked a series of questions about the experiment prior to being fully debriefed. The entire procedure lasted approximately 40 min.

### Results

No participant correctly guessed the main manipulation and purpose of the experiment without prompting from the experimenter. Details of the responses to the postexperimental questions can be found in Appendix J. We first discuss the results for the HV and VV tasks which measured perceived object size, followed by the results for grasping capacity.

#### Perceived object size

Fourteen individual trials were removed (2 HV-left, 2 HV-right, 6 VV-left, and 4 VV-right) where invalid responses occurred (e.g., pressing the spacebar without adjusting the distance of the lines). Ratios were then calculated for the visually estimated size of each block by dividing the estimated size by the actual size. Linkenauger et al. (2011) claimed that action-specific scaling effects should only occur when the action in question is performable. Therefore, to be consistent with Linkenauger et al. (2011), here we report the analysis only for the average ratio for trials where graspable stimuli were presented, based on the largest block that participants were able to grasp in the grasping capacity task (we also report results for the average ratio of all 21 sizes in Appendix A).

**Figure 2 fig2:**
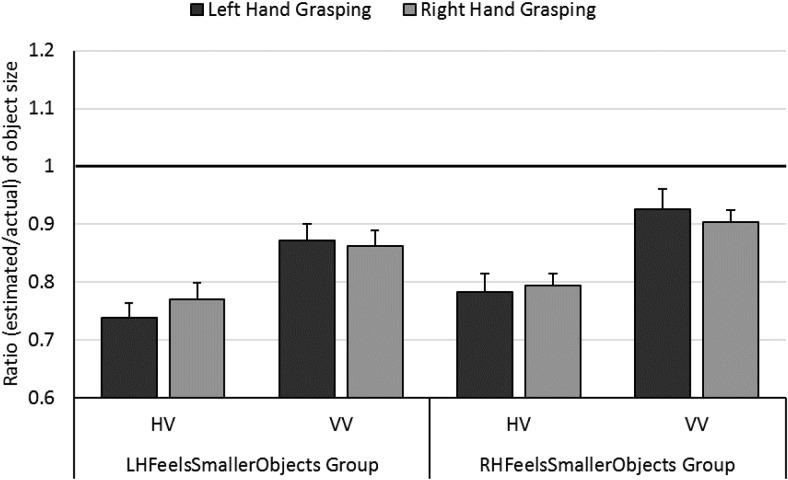
Results of the object size estimation Task in Experiment 2: Size estimates of objects grasped in the left and right hands in the HV and VV tasks for the LHFeelsSmallerObjects and RHFeelsSmallerObjects Prime Groups. A ratio of 1 (highlighted in bold) represents perfect accuracy. One-sample *t* tests on ratios for the LHFeelsSmallerObjects Prime Group for the HV-left, HV-right, VV-left and VV-right conditions were all significantly lower than 1, *t*(14) = −10.01, *t*(14) = −7.70, *t*(14) = −3.40, and *t*(14) = −4.02, respectively, all *p* < .001. Similarly, for the RHFeelsSmallerObjects Prime Group, ratios for HV-left, HV-right, VV-left and VV-right conditions were all significantly lower than 1, *t*(14) = −7.40, *t*(14) = −7.77, *t*(14) = −3.50, and *t*(14) = −4.61, respectively, all *p* < .001. Error bars show ± one standard error of the mean.

A mixed ANOVA was conducted where Grasping Hand (left/right) and Task (HV/VV) were within-participants factors and Prime Group (LHFeelsSmallerObjects/RHFeelsSmallerObjects) was a between-participants factor (*p* values for pairwise comparisons were Bonferroni corrected). This revealed that ratios for the left hand grasping (0.82) did not differ significantly from ratios for the right hand grasping (0.83), *F*(1, 28) = 0.27, *p* = .6, η_p_^2^ = .01, see Figure 2. Ratios were significantly greater in the VV Task (0.89) than in the HV Task (0.77), *F*(1, 28) = 49.37, *p* < .001, η_p_^2^ = .64, so people underestimated size more when the blocks were perceived haptically rather than visually. There was no significant effect of Prime Group, *F*(1, 28) = 1.42, *p* = .2, η_p_^2^ = .05, of Task × Prime Group, *F*(1, 28) = 0.17, *p* = .7, η_p_^2^ = .01, of Grasping Hand × Prime Group, *F*(1, 28) = 2.16, *p* = .2, η_p_^2^ = .07, or of Grasping Hand × Task × Prime Group, *F*(1, 28) = 0.16, *p* = .7, η_p_^2^ = .004. The only significant interaction was for Grasping Hand × Task, *F*(1, 28) = 9.75, *p* = .004, η_p_^2^ = .26. There is some evidence that for touch, contrary to the predictions of the action-specific account, objects may feel larger if the hand is perceived as larger (Bruno & Bertamini, 2010). Consistent with this proposal, pairwise comparisons showed that ratios were significantly greater for the right hand grasping in the HV Task (mean difference = 0.021, *p* = .011). In the VV Task, ratios for the right hand were not significantly lower, as the action-specific account would predict, though the trend was in this direction (mean difference = −0.015, *p* = .08).[Fig-anchor fig2]

We ran a Bayesian analysis to check the strength of evidence for the null effects revealed by the ANOVA, see Table 1. We used the procedure described by Masson (2011) and the descriptive terms for strength of evidence suggested by Raftery (1995).[Table-anchor tbl1]

#### Actual grasping capacity

A mixed ANOVA analyzing the maximum grasp for each Grasping Hand (left/right) as a within-participants factor and Prime Group (LHFeelsSmallerObjects/RHFeelsSmallerObjects) as a between-participants factor was conducted. This revealed no effect of Grasping Hand, *F*(1, 28) = 0.33, *p* = .6, η_p_^2^ = .01, Prime Group, *F*(1, 28) = 1.22, *p* = .3, η_p_^2^ = .04, or a Grasping Hand × Prime Group interaction, *F*(1, 28) = 1.32, *p* = .3, η_p_^2^ = .05. Thus, although Experiment 1 found that most right-handed observers think that their right hand is larger than their left, we found no evidence in Experiment 2 that the right hand actually has a greater grasping capacity than the left hand.

### Discussion

In Experiment 2 we tested whether perceived differences in grasping capacity would influence the perceived size of objects presented either visually or haptically. There were two reasons why objects grasped by the right hand might be perceived as smaller than objects grasped by the left hand: first, a preexisting tendency for right-handers to overestimate the size of their right hand (Linkenauger et al., 2009; replicated in Experiment 1 here) which could lead to them overestimating the grasping capacity of their right hand (Linkenauger et al., 2011); and, second, a priming manipulation intended to make observers feel that their right hand had a greater grasping capacity by having it grasp a set of smaller objects than the left hand before estimates were made. We also tested whether being primed to feel that the left hand had a greater grasping capacity would reduce estimates of object size for objects grasped in the left hand.

Our results suggest that neither our priming manipulation nor a preexisting overestimation of the grasping capacity of the right hand influenced visually perceived object size. We thus found no action-specific scaling effect for visually presented stimuli. The only effect we found was that, for the HV Task, objects grasped in the right hand were estimated as slightly larger than objects grasped in the left hand, regardless of priming group. This result is consistent with findings that unseen stimuli are estimated as larger if they are felt by a body part which is perceived as larger (Bruno & Bertamini, 2010; Taylor-Clarke et al., 2004). This latter, size-scaling effect was in the opposite direction to that predicted by the action-specific account, so we suggest that it does not reflect perceptual scaling based on perceived grasping capacity. Instead, this effect may reflect a difference in the perceived size of the left and right hands, consistent with the results of Experiment 1. This effect may arise from the greater representation in the somatosensory cortex of the right than the left hand for right handers (Sörös et al., 1999). This implies that the right hand may have smaller receptive fields and be more sensitive to touch than the left hand, causing unseen objects held in the right hand to be estimated as larger. This suggests that the acuity of touch may influence visual estimates of object size as a result of multimodal integration. In summary, Experiment 2 suggested that perceived object size was not influenced in ways predicted by the action-specific account.

## Experiment 3

Experiment 1 demonstrated that right-handed observers perceived their right hand as larger than their left hand, whereas Experiment 2 suggested that this asymmetry in perceived hand size does not lead to differences in perceived object size when attention is not explicitly drawn to action capacity. However, we did not measure perceived maximum grasp in Experiment 2 so we could not be certain that our hand dominance and priming manipulations of perceived action capacity were effective. To address this point, in Experiment 3 we used the VV task from Experiment 2 to measure perceived object size and then, afterward, we measured perceived maximum grasping capacity for each hand.

### Method

#### Participants

Thirty right-handed undergraduate students from the University of Liverpool (22 females, mean age = 19.9 years, mean Edinburgh Handedness Inventory score = 85, range = 37.5–100) were recruited for this experiment. Participants either volunteered or were rewarded with course credit.

#### Stimuli, design, and procedure

The stimuli, design, and procedure were identical to Experiment 2, except that there was no HV task and perceived maximum grasping capacity for each hand was measured after completion of the VV task. Here, participants were asked which block they believed was the largest they could grasp (again, using their thumb on one side and any other finger on the opposite side) in each hand. Participants saw nine foam board blocks, 0.5 cm deep, which were laid out in size order on a shelf from 14 cm (on the far left) to 22 cm (on the far right), in 1 cm increments. Participants pointed at the block that they believed was the biggest one they could grasp.

### Results

No participant correctly guessed the main manipulation and purpose of the experiment without prompting from the experimenter. Details of the responses to the postexperimental questions can be found in Appendix J. We first discuss the results for the VV Task which measured perceived object size, followed by the results for perceived and for actual grasping capacity.

#### Perceived object size

Ratios were calculated for each block as in Experiment 2. For consistency with Linkenauger et al. (2011), here we report only the results for stimuli that participants perceived they could grasp (results for the full dataset are reported in Appendix B, and results based on whether participants could actually grasp the stimuli are reported in Appendix C).

**Figure 3 fig3:**
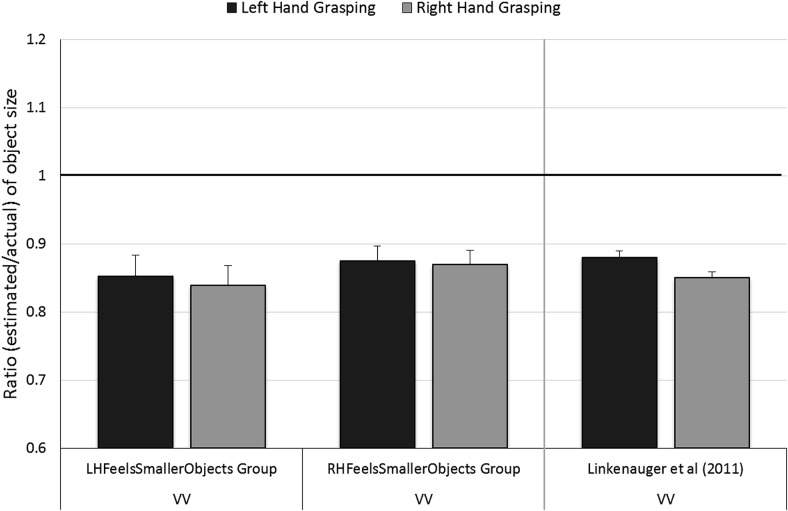
Results of the object size estimation Task in Experiment 3: Size estimates of objects grasped in the left and right hands in the VV Task for the LHFeelsSmallerObjects and RHFeelsSmallerObjects Prime Groups. For comparison, we include data from Linkenauger et al. (2011). A ratio of 1 (highlighted in bold) represents perfect accuracy. One-sample *t* tests showed that ratios for the VV-left and VV-right conditions were both significantly lower than 1 for the LHFeelsSmallerObjects group, *t*(14) = −4.89, and *t*(14) = −5.69, respectively, and for the RHFeelsSmallerObjects group, *t*(14) = −5.97, and *t*(14) = −6.40, respectively, all *p* < .001. Error bars show ± one standard error of the mean.

A mixed ANOVA with Grasping Hand (left/right) as a within-participants factor and Prime Group (LHFeelsSmallerObjects/RHFeelsSmallerObjects) as a between-participants factor was conducted. This revealed no significant effects of Grasping Hand, *F*(1, 28) = 1.70, *p* = .2, η_p_^2^ = .06, Prime Group, *F*(1, 28) = 0.39, *p* = .5, η_p_^2^ = .01, or of Grasping Hand × Prime Group, *F*(1, 28) = 0.20, *p* = .7, η_p_^2^ = .004, see Figure 3.[Fig-anchor fig3]

As in Experiment 2, we ran a Bayesian analysis to check the strength of evidence for the null effects revealed by the ANOVA, see Table 2.[Table-anchor tbl2]

#### Actual and perceived grasping capacity

**Figure 4 fig4:**
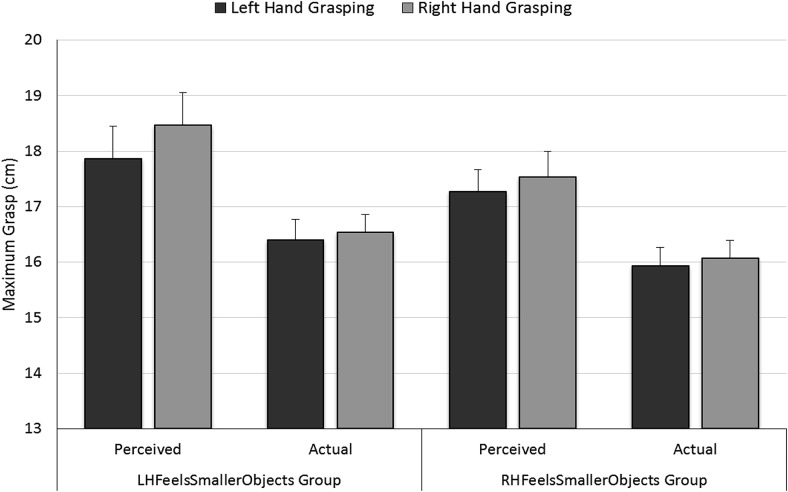
Results of the maximum grasping capacity tasks in Experiment 3: Estimates of maximum grasp for the left and right hands for the LHFeelsSmallerObjects and RHFeelsSmallerObjects groups. Perceived grasp is the largest block participants believed they could grasp. Actual grasp is the largest block that could be grasped in each hand. Error bars represent ± one standard error of the mean.

We analyzed participant’s perceived and actual maximum grasp for their left and right hands in separate^1^ mixed ANOVAs where Hand (left/right) was a within-participants factor and Prime Group (LHFeelsSmallerObjects/RHFeelsSmallerObjects) was a between-subjects factor. For *perceived grasp*, maximum grasp for the right hand (18.0 cm) was greater than for the left hand (17.5 cm), *F*(1, 28) = 10.85, *p* = .003, η_p_^2^ = .30, see Figure 4. There was no significant effect of Prime Group, *F*(1, 28) = 1.15, *p* = .3, η_p_^2^ = .04, or of Hand × Prime Group, *F*(1, 28) = 1.61, *p* = .2, η_p_^2^ = .05. For *actual grasp*, maximum grasp for the right hand (16.2 cm) did not differ from the left hand (16.3 cm), *F*(1, 28) = 1.11, *p* = .3, η_p_^2^ = .04. There was no significant effect of Prime Group, *F*(1, 28) = 1.01, *p* = .3, η_p_^2^ = .04, or of Hand × Prime Group, *F*(1, 28) = 0.001, *p* = .9, η_p_^2^ < .001. Together these results suggest that these right-handed participants estimated the maximum grasp of their right hand as greater than that of their left (replicating Experiment 1) but that there was no difference in the actual grasping capacities of their hands (replicating Experiment 2)[Fig-anchor fig4].

### Discussion

In Experiment 3 we showed that, regardless of prime group, participants overestimated the grasping capacity of their right hand relative to their left hand. This supports the findings of Experiment 1 which showed that right handers usually think their right hand is larger than their left hand. Nevertheless, replicating the results of Experiment 2, neither a preexisting overestimation of the grasping capacity of the right hand, nor our priming manipulation influenced estimates of object size. Thus although participants overestimated the grasping capacity of their right hand relative to their left hand, this did not influence their perception of object size when no attention was drawn to action. Together these results suggest that perceived object size is not directly influenced by perceived action capacity.

## Experiment 4

In Experiments 2 and 3 we found no evidence that overestimating the grasping ability of one hand relative to the other has a direct influence on visual perceptions of object size. Consistent with Linkenauger et al. (2009, 2011), in Experiment 1, right-handers *perceived* their right hand as larger than their left, and in Experiment 3 participants *perceived* the grasping capacity of their right hand as larger than their left hand. However, this latter effect was modest, with the right hand estimated as being able to grasp objects that were, on average, only 0.5 cm larger. Furthermore, Experiment 3 showed that there was no difference in the *actual* grasping capacity of the right and left hands, replicating Linkenauger et al. (2011). Finally, our priming manipulation in Experiment 3 did not influence the relative perceived grasping capacity of the hands. This might be because the effect of priming dissipated during the size estimation Task and thus was not detected in the subsequent grasping capacity Task. Here, if acting rapidly recalibrates perceived action capacity (Franchak & Adolph, 2014) then grasping during the VV Task may have overridden any changes in perceived grasping capacity from the priming manipulation.

Thus it is possible that in Experiments 2 and 3 we found no scaling effects on perceived object size that were consistent with the action-specific account because there was only a modest difference in the *perceived* grasping capacity of the left and right hands, or because there was no difference in the *actual* grasping capacity of the left and right hands. Related to this second point, Sugovic et al. (2016) found that only actual differences in body size, and not people’s beliefs or perceptions about their body size, affected spatial perception. To examine both of these possibilities, in Experiment 4 we used a more powerful taping manipulation which produced substantial changes in actual as well as perceived grasping capacity.

Surprisingly, comparisons between conditions where the spatial extent to be estimated is kept constant but action capacity is varied have rarely been reported in the action-specific literature, although some manipulations which alter the action boundaries of the body have been previously shown to influence spatial perception (Lessard, Creem-Regehr, & Stefanucci, 2012; Witt et al., 2005). One such study was conducted by Shaffer and Flint (2011), who showed that estimated slant for an escalator did not differ to estimated slant for a set of stairs. This is inconsistent with the action-specific account which suggests that the stairs should have appeared steeper because they require effort to climb, unlike standing on an escalator. In Experiment 4 here, to directly alter participants’ ability to grasp objects, we taped together the fingers of one hand to reduce its grasping capacity relative to the untaped hand. We predicted that participants would estimate their maximum grasp to be lower when their hand was taped relative to when it was untaped. Nevertheless, based on the results of Experiments 2 and 3, we predicted that even if there was a large change in perceived (and actual) action capacity following taping this would not alter estimates of object size.

### Method

#### Participants

Thirty right-handed undergraduate students (26 females, mean age = 20.1 years, mean Edinburgh Handedness Quotient score = 81, range = 50–100) were recruited from the University of Liverpool. Participants either volunteered or received course credit for their time.

#### Stimuli, apparatus, and procedure

The stimuli, apparatus, and procedure were identical to Experiment 3 apart from the following changes. First, we included the HV task from Experiment 2. Second, instead of the priming phase, the fingers were taped on either the left hand (LHTaped group) or the right hand (RHTaped group). The middle and ring fingers were first taped together above the proximal interphalangeal (middle) finger joint, then all four fingers were taped together just under the same joint. The hand remained taped while participants completed the HV and then the VV tasks. After completing these two object size estimation tasks, participants’ perceived maximum grasp followed by actual maximum grasp were measured for the untaped hand, then for the taped hand, and finally for the taped hand after removing the tape. The postexperimental questions were similar to those asked in Experiments 2 and 3, but were reworded to better fit the taping manipulation.

### Results

No participant correctly guessed the main manipulation and purpose of the experiment without prompting from the experimenter. Details of the responses to the postexperimental questions can be found in Appendix J. We first discuss the results for the HV and VV tasks which measured perceived object size, followed by the results for perceived and actual grasping capacity.

#### Perceived object size

Ratios were calculated for each block as in Experiments 2 and 3. For consistency with Linkenauger et al. (2011), here we report only the results for stimuli that participants perceived they could grasp (results for the full dataset are reported in Appendix D, and results based on whether participants could actually grasp the stimuli are reported in Appendix E).

**Figure 5 fig5:**
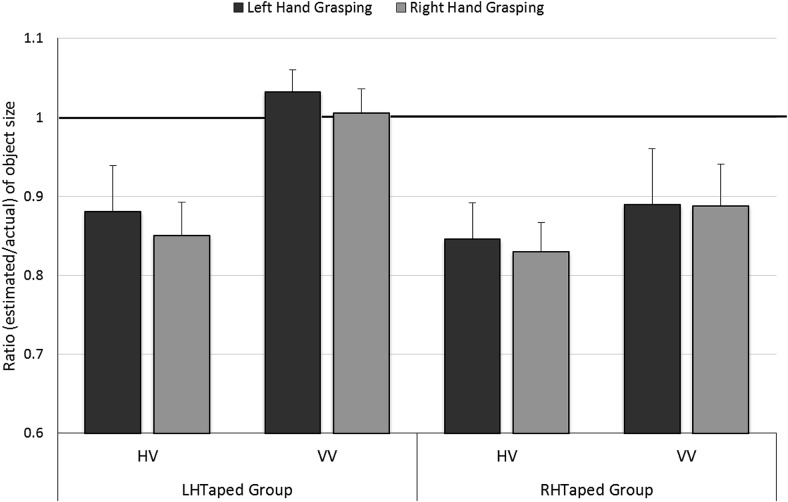
Results of the object size estimation Task in Experiment 4: Size estimates of objects grasped in the left and right hands in the HV and VV tasks for the LHTaped and RHTaped Tape Groups. A ratio of 1 (highlighted in bold) represents perfect accuracy. One-sample *t* tests showed that for the LHTaped group, the HV-left and HV-right conditions were significantly lower than 1, *t*(14) = −2.61, *p* = .02, and *t*(14 = −4.03, *p* = .001, respectively), whereas ratios for the VV-left and VV-right conditions did not differ from 1, *t*(14) = 0.45, *p* = .7, and *t*(14) = 0.07, *p* = .9, respectively. For the RHTaped group, ratios for the HV-left, HV-right, VV-left and VV-right conditions were all significantly lower than 1, *t*(14) = −4.00, *t*(14) = −3.99, *t*(14) = −3.84, and *t*(14) = −3.83, respectively, all *p* < .001. Error bars show ± one standard error of the mean

A mixed ANOVA with Grasping Hand (left/right) and Task (HV/VV) as within-participants factors and Tape Group (LHTaped/RHTaped) as a between-participants factor was conducted, see Figure 5. Importantly, no significant effect was found for Grasping Hand *F*(1, 28) = 0.33, *p* = .6, η_p_^2^ = .01. As in Experiment 2, ratios were significantly lower for the HV Task (0.84) than the VV Task (0.95), *F*(1, 28) = 16.28, *p* < .001, η_p_^2^ = .37, so people underestimated size more when the blocks were perceived haptically rather than visually. There were no other significant effects: Tape Group, *F*(1, 28) = 2.42, *p* = .1, η_p_^2^ = .08; Task × Tape Group, *F*(1, 28) = 2.44, *p* = .1, η_p_^2^ = .08; Grasping Hand × Tape Group, *F*(1, 28) = 1.10, *p* = .3, η_p_^2^ = .04; Task × Grasping Hand, *F*(1, 28) = 0.11, *p* = .7, η_p_^2^ = .01; Grasping Hand × Task × Tape Group, *F*(1, 28) = 0.28, *p* = .6, η_p_^2^ = .01.[Fig-anchor fig5]

As in Experiments 2 and 3, we ran a Bayesian analysis to check the strength of evidence for the null effects revealed by the ANOVA, see Table 3.[Table-anchor tbl3]

#### Actual and perceived grasping capacity

We analyzed participants’ perceived and actual maximum grasp for their left and right hands in separate^2^ ANOVAs, where Grasping Hand (left/right) was a within-participants factor and Tape Group (LHTaped/RHTaped) was a between-participants factor (*p* values for pairwise comparisons were Bonferroni corrected). For *perceived grasp* there was no significant effect of Grasping Hand, *F*(1, 28) = 0.51, *p* = .8, η_p_^2^ = .02, or of Tape Group, *F*(1, 28) = 1.52, *p* = .2, η_p_^2^ = .05, but there was a Grasping Hand × Tape Group interaction, *F*(1, 28) = 118.37, *p* < .001, η_p_^2^ = .81. Pairwise comparisons showed that, as expected, perceived maximum grasp was smaller for the left hand (14.9 cm) than the right hand (18.1 cm) in the LHTaped group (mean difference = −3.3 cm, *p* < .001), but was larger for the left hand (17.5 cm) than the right hand (14.4 cm) in the RHTaped group (mean difference = 3.1 cm, *p* < .001). Thus both groups appropriately recalibrated their estimates of maximum grasp following taping of their hand.

**Figure 6 fig6:**
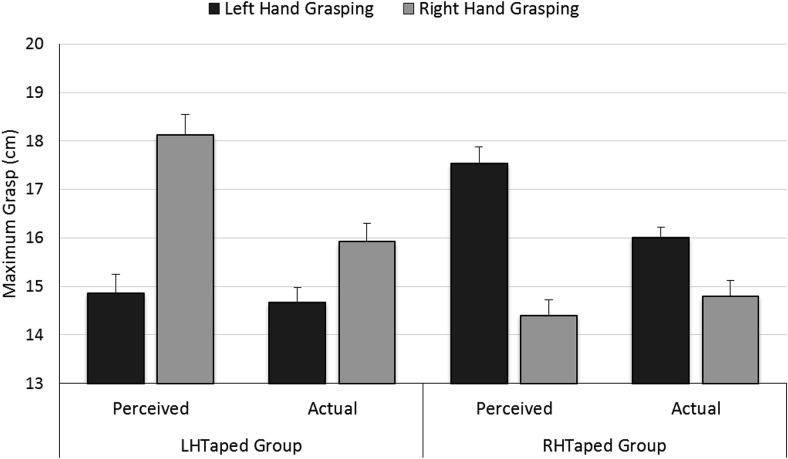
Results of the maximum grasping capacity tasks in Experiment 4: Estimates of maximum grasp for the left and right hands for the LHTaped and RHTaped groups. Perceived grasp is the largest block participants believed they could grasp. Actual grasp is the largest block that they could, in fact, grasp. Error bars represent ± one standard error of the mean.

Similarly, for *actual grasp*, there was no significant effect of Grasping Hand, *F*(1, 28) = 0.40, *p* = .8, η_p_^2^ = .01, or of Tape Group, *F*(1, 28) = 0.06, *p* = .8, η_p_^2^ = .02, but Grasping Hand × Tape Group was again significant, *F*(1, 28) = 54.76, *p* < .001, η_p_^2^ = .66. Pairwise comparisons showed that actual maximum grasp was smaller for the left hand (14.7 cm) than the right hand (15.9 cm) in the LHTaped group (mean difference = −1.3 cm, *p* < .001), but was larger for the left hand (16.0 cm) than the right hand (14.8 cm) in the RHTaped group (mean difference = 1.2 cm, *p* < .001). Thus, for both groups, taping a hand reduced the size of the biggest block it could actually grasp, with a similar, but enhanced, pattern of effects on perceived grasp, see Figure 6.[Fig-anchor fig6]

### Discussion

In Experiment 4, taping one hand reduced estimates of the perceived maximum grasping capacity of that hand by, on average, 3.2 cm, and actual grasp by 1.2 cm, relative to the untaped hand (see Figure 6). This powerful manipulation had, though, no influence on estimates of object size (see Figure 5). Experiments 2 and 3 found no evidence that a difference in perceived grasping capacity (either due to right-hand dominance or to priming) influenced perceived object size. Experiment 4 provided direct experimental evidence supporting this finding. Participants rapidly and appropriately recalibrated their perceived action capacity when their fingers were taped together (see also Franchak & Adolph, 2014; Ishak et al., 2008). However, this recalibration had no impact on perceived object size. We also found no evidence that actual grasping capacity influences perceived object size.

## Experiment 5

Together, the results of Experiments 2 through 4 suggest that grasping capacity does not directly influence perceived object size. This conclusion is not consistent with Linkenauger et al. (2011), who concluded that because right handers perceive their right hand as having a greater grasping capacity then objects grasped by the right hand are perceived as smaller than those grasped by the left hand. However, the method used in our experiments deviated in a number of ways from the experiments of Linkenauger et al. (2011). For example, because the blocks were placed under the monitor, participants may have tried to use landmark matching to make their estimates (though some evidence suggests that people do not spontaneously use landmark matching, e.g., Lawson & Bertamini, 2006, Experiment 4, and note that the blocks were offset from the response lines, see Figure 1A).

Arguably the most important change made was that we did not ask participants about their grasping capacity on each size estimation trial. As discussed in the introduction, we reasoned that if intending to act is sufficient to induce action-specific scaling effects, then scaling effects should occur if participants actually grasp an object and not only when they imagine grasping it. Actual grasping preceded every size estimation trial in Experiments 2 through 4 and yet we found no action-specific scaling effects. We made this change because of our concern that the results reported by Linkenauger et al. (2011) could have arisen because imagining grasping an object to verbally report its graspability may have drawn attention to action. This may have led their participants to conflate estimates of action capacity (graspability) with their subsequent estimates of object size. If so, then their participants may not have experienced a change in perceived object size in the strongest sense. However, it could be argued that, in Experiments 2 through 4, we not only removed this potential conflation but that we also removed participants’ intention to act on the object that they were estimating the size of. This is because our participants had finished picking up and moving the object before they estimated its size, and they did not act on it again until after making their estimates. It is also possible that because grasping is such an everyday action, participants were not thinking about the action in a way which made it seem relevant to the task. We tackled these possibilities, and others, in Experiment 5 by moving to a method more similar to that used by Linkenauger et al. (2011), as described below.

Importantly, though, we wanted to still ensure that any effects that we observed could not be attributed to demand characteristics, which has been a concern with the action-specific account (for reviews see Firestone, 2013; Firestone & Scholl, 2014, 2015; Philbeck & Witt, 2015), or conflation. Demand characteristics refer to participants altering their behavior in accordance with what they believe the experimenter’s hypothesis to be. A number of studies have tried to control for demand characteristics, for example by assessing individual differences (Linkenauger et al., 2011, Experiment 3) or by using indirect measures (Witt, 2011b). However, there is evidence that when demand characteristics are reduced, for example by giving participants a cover story for an otherwise unexplained manipulation, action-specific effects may disappear. For example, Durgin et al. (2009; see also Durgin, Klein, Spiefel, Strawser, & Williams, 2012; Shaffer, McManama, Swank, & Durgin, 2013) showed that participants wearing a heavy backpack did not estimate slant as steeper than those who did not wear a backpack when they were told that the backpack contained equipment that monitored their ankle muscles. This suggests that when no explanation is provided for wearing the backpack, participants may infer that wearing it is intended to increase their estimates of slant, and so they might adjust their estimates accordingly.

Thus, in Experiment 5, although we explicitly told participants that we were interested in graspability, and although on every trial they actually grasped the object both before and after estimating its size, we used a cover story to minimize the chances of finding an effect simply due to conflation or demand characteristics. Specifically, we told participants that, for practical reasons, we were running two separate studies simultaneously, one of which was a grasping task and the other was a size matching task. They were told that the experimenter would record how they grasped each object to provide data for a control study about grasping behavior which was independent of the main experiment in which they estimated object size. We also provided a cover story for the postestimation grasp by asking participants to hand the object back to the experimenter after making their size estimate. Finally, we made a number of further changes (such as removing the priming and HV tasks, blocking rather than randomizing trials with each hand, and having the non-action-relevant hand make responses) to further reduce the differences between our previous experiments and those of Linkenauger et al. (2011).

In Experiment 5 we manipulated perceived graspability using the same, direct manipulation of hand taping that we used in Experiment 4, as well as using the preexisting effect of right-hand dominance used in Experiments 2 and 3. We minimized conflation effects by using a cover story and tested whether our previous results were attributable to participants in Experiments 2 through 4 not thinking about grasping when they estimated object size. If the results of Experiment 5 show an effect of perceived grasping capacity on estimated object size, this would replicate the findings of Linkenauger et al. (2011) and suggest that the lack of an immediate intention to act may be the critical difference between their studies and Experiments 2 through 4 here. However, if the results showed no such effect, it would suggest that intention to grasp is not sufficient to scale perceived object size. This, in turn, would suggest that Linkenauger et al.’s (2011) results may have arisen because their participant’s attention was drawn to the possible association between grasping capacity and object size, and not due to cognitive penetrability resulting in perceptual scaling in the strongest sense (Firestone & Scholl, 2015).

### Method

#### Participants

Thirty-two (24 females, mean age = 19.3 years, mean Edinburgh Handedness Inventory score = 91, range = 62.5–100) right-handed participants were recruited for this study. Participants either volunteered or were rewarded with course credit for their time.

#### The instructions

In Experiment 5, we ensured that participants were thinking about grasping by informing them at the beginning of the experiment that we were interested in both how they grasped the blocks and how well they could visually match the size of the blocks. They were told that they would do two separate studies during the same session, due to time constraints in data collection. We also told them that they would have to hand the blocks back to the experimenter after making their size estimates. The full instructions are given in Appendix F.

#### Apparatus, stimuli, and procedure

The apparatus, stimuli, and procedure were identical to Experiment 4 apart from the following changes. We used a laptop (monitor = 27 × 35 cm) which was placed at 90° to the participant. The laptop was placed on the opposite side to the grasping hand used for that block and, to be consistent with Linkenauger et al. (2011), participants responded with the nongrasping hand. For example, if they grasped the block with their left hand, the laptop was placed on their right hand side and they responded with their right hand. Responses were made using the up and down arrow keys on the laptop keyboard (to move the lines further apart and closer together respectively). Trials were blocked by hand. There was no HV task and, instead, participants completed three subblocks of the VV task. In one subblock, they grasped the blocks with their untaped left hand and estimated size with their untaped right hand, and in a second subblock the assignment of task to hands was reversed. In the third subblock, participants had the fingers of one hand taped as in Experiment 4 and they grasped blocks with their taped hand and responded with their untaped hand. Half of the participants completed the first two subblocks in each order and, of these, half had their left hand taped (LHTaped Group) and half had their right hand taped (RHTaped group) in the final subblock.

The box was removed so participants saw the blocks before they grasped them. The experimenter checked whether participants performed the specified grasp on each trial, see Appendix G. If participants did not initially attempt the specified grasp, they were reminded to do so by the experimenter. If the object was too big to be successfully grasped in this way, the experimenter recorded how the participant then chose to pick up the object (e.g., by the corner). Participants completed 63 VV trials (21 stimuli × 3 subblocks) and the whole procedure lasted around 30 min.

### Results

One participant correctly guessed the aims and purpose of the experiment during the postexperimental questions (after question 3) but their data are still included in the analysis. As has been done in previous work investigating participants’ beliefs (e.g., Durgin et al., 2012), we provide responses to the postexperimental questions in Appendix K. In this section, we first discuss the results for the VV task which measured perceived object size, followed by the results for perceived and actual grasping capacity.

#### Perceived object size

Ratios were calculated for each block, as in Experiments 2 through 4. For consistency with Linkenauger et al. (2011), here we report only the results for stimuli that participants perceived they could grasp (results for the full dataset are reported in Appendix H, and results based on whether participants could actually grasp the stimuli are reported in Appendix I).

**Figure 7 fig7:**
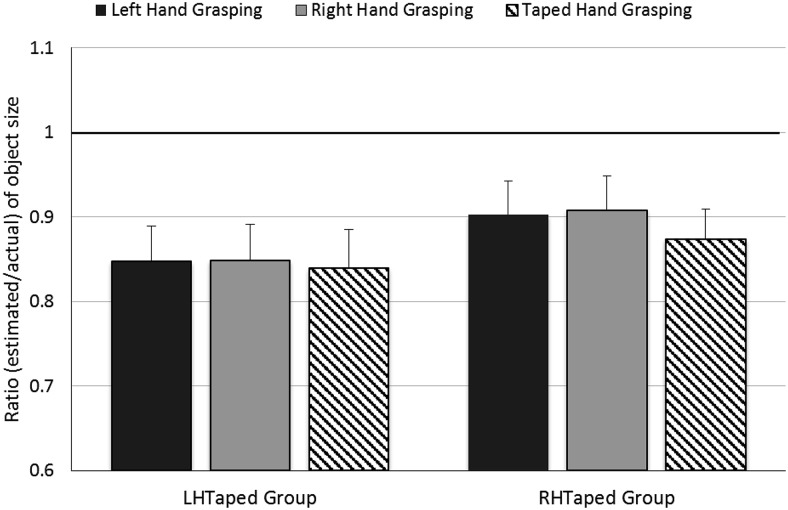
Results of the object size estimation task in Experiment 5: Size estimates of objects grasped in the left, right and taped hands for the LHtaped and RHTaped groups. A ratio of 1 (highlighted in bold) represents perfect accuracy. One-sample *t* tests showed that for the LHTaped group, estimates for the left, right and taped hands were all significantly lower than 1, *t*(15) = −3.85, *p* = .002, *t*(15) = −3.77, *p* = .002, and *t*(15) = −4.47, *p* < .001, respectively. For the RHTaped group, estimates for the left and taped hands were significantly lower than 1, *t*(15) = −2.32, *p* = .035, and *t*(15) = −2.74, *p* = .015, respectively, and estimates for the right hand were marginally lower than 1, *t*(15) = −2.13, *p* = .05. Error bars show ± one standard error of the mean.

A mixed ANOVA with Grasping Hand (left/right/taped) as a within-participants factor and Tape Group (LHTaped/RHTaped) as a between-participants factor was conducted. This revealed that neither Grasping Hand, *F*(2, 60) = 0.48, *p* = .6, η_p_^2^ = .02, nor Tape Group, *F*(1, 30) = 0.95, *p* = .4, η_p_^2^ = .03, influenced estimated object size, and there was no Grasping Hand × Tape Group interaction, *F*(2, 60) = 0.16, *p* = .9, η_p_^2^ = .01 (see Figure 7).[Fig-anchor fig7]

As in Experiments 2 through 4, we ran a Bayesian analysis to test the strength of evidence for the null effects revealed by the ANOVA (see Table 4).[Table-anchor tbl4]

#### Actual and perceived grasping capacity

**Figure 8 fig8:**
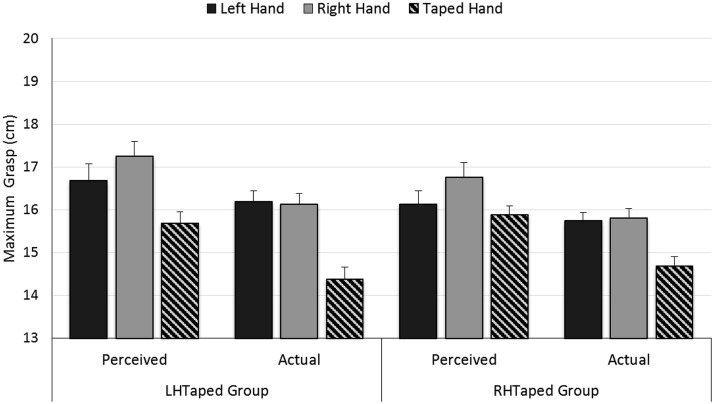
Results of the maximum grasping capacity tasks in Experiment 5: Estimates of maximum grasp for the left and right hands for the LHTaped and RHTaped groups. Perceived grasp is the largest block participants believed they could grasp. Actual grasp is the largest block that could, in fact, be grasped. Error bars represent ± one standard error of the mean.

We analyzed participant’s perceived and actual maximum grasp for their left and right hands in separate mixed ANOVAs where hand (left/right/taped) was a within-participants factor and Tape Group (LHTaped/RHTaped) was a between-subjects factor.^3^ For *perceived grasp*, the right hand (17.0 cm) was perceived as having a greater grasping capacity than both the left hand (16.4 cm) and the taped hand (15.8 cm), *F*(2, 60) = 20.50, *p* < .001, η_p_^2^ = .41 (see Figure 8). There was no effect of Tape Group, *F*(1, 30) = 0.60, *p* = .5, η_p_^2^ = .02, nor a Hand × Tape Group interaction, *F*(2, 60) = 2.39, *p* = .1, η_p_^2^ = .07. For *actual grasp*, the right (16.0 cm) and left (16.0 cm) hands did not differ, but the taped hand was significantly less (14.5 cm), *F*(2, 60) = 73.47, *p* < .001, η_p_^2^ = 0.71. There was no effect of Tape Group, *F*(1, 30) = 0.24, *p* = .6, η_p_^2^ = .01, but there was a Hand × Tape Group interaction, *F*(2, 60) = 4.306, *p* = .018, η_p_^2^ = .13. For both groups, taping reduced actual maximum grasp relative to both hands, with this reduction being somewhat larger for the LHTaped group (mean 1.8 cm) than the RHTaped group (mean 1.1 cm).[Fig-anchor fig8]

### Discussion

In Experiment 5, we eliminated a number of methodological differences between Experiments 2 through 4 reported here and the experiments reported by Linkenauger et al. (2011) to test whether these differences could explain why Linkenauger et al. found an effect of grasping capacity on perceived object size but we did not. Most importantly, we changed our instructions so participants were explicitly told that we were interested in whether they could grasp each object using their thumb and finger. In addition, we changed the trial procedure so that participants always intended to act on the object when they were estimating its size, by having them pick up the object to give it back to the experimenter after making their size estimate. Nevertheless, we replicated our findings from Experiment 4. Specifically, although participants believed they could grasp larger objects with their right compared with their left hand, and with their untaped rather than their taped hand (see Figure 8), neither of these effects on perceived action capacity modulated their estimates of object size (see Figure 7).

Participants in Experiment 5 both intended to act, and did indeed act, on a given object both before and after estimating the size of that object, and they were explicitly and repeatedly told that we were assessing both their grasping capacity and their estimates of object size. However, we provided a cover story to persuade participants that there was no relation between our interest in their grasping capacity and in their object size estimates (only one participant guessed the true purpose of the study). Therefore, Linkenauger et al.’s (2011) results may have reflected a conflation of estimates of perceived grasping capacity and of object size which arose from asking participants about both action capacity and object size on each trial without providing any explanation of why both measures were being taken (Collier & Lawson, 2017).

## General Discussion

The action-specific account of perception suggests that an observer’s action capacity scales how they perceive the spatial properties of the environment (Bhalla & Proffitt, 1999; Fajen, 2005; Linkenauger et al., 2009, 2010, 2011; Proffitt et al., 1995, 2003; Proffitt, 2006a, 2006b, 2013; Proffitt & Linkenauger, 2013; Witt et al., 2004, 2005; Witt, 2011a; Witt & Riley, 2014). However, other evidence suggests that estimates of spatial attributes, such as distance, may only scale according to action capacity when observers are encouraged to consider nonvisual factors (Woods et al., 2009; Firestone, 2013).

In five experiments, we tested whether the perceived size of graspable objects is scaled according to people’s ability to pick up those objects. We began by testing the claim that right-handed individuals overestimate the grasping capacity of their right hand relative to their left hand and, because of this, they underestimate the size of objects to-be-grasped in their right hand (Linkenauger et al., 2009, 2011). We replicated the finding that right handers perceive their right hand as both larger (Experiment 1) and as having a greater grasping capacity (Experiments 3 and 5) than their left hand. In addition, when the fingers of one hand were taped together, participants appropriately reduced their estimates of the maximum grasp of that hand (Experiments 4 and 5). However, none of our three manipulations of perceived grasping capacity—right hand dominance (Experiments 2, 3, and 5), priming (Experiments 2 and 3) and restricting the grasp of the hand by taping (Experiments 4 and 5)—reliably modulated estimates of object size, whether objects were presented visually (for the VV tasks) or haptically (for the HV tasks). Thus we did not replicate the results of Linkenauger et al. (2011) because we failed to find the predicted influence of perceived action capacity on spatial perception.

The exact relationship between spatial properties and perceived action capacity is not yet fully understood (Cañal-Bruland & van der Kamp, 2015). Nevertheless, our results are consistent with previous work demonstrating that estimates of spatial features are not always predicted by perceived action capacity (e.g., De Grave, Brenner, & Smeets, 2011; Woods et al., 2009). For example, Cañal-Bruland, Aertssen, Ham, and Stins (2015) failed to replicate the reported finding that decreasing postural stability makes walkable beams appear narrower (Geuss, Stefanucci, de Benedictis-Kessner, & Stevens, 2010). Other studies have shown that providing a cover story for otherwise unexplained task manipulations can offset action-specific scaling effects (e.g., Durgin et al., 2009; Firestone & Scholl, 2014; we used a similar manipulation in Experiment 5 here). For example, Firestone and Scholl (2014) showed that apertures are not estimated as narrower while holding a rod that is wider than the body (as reported by Stefanucci & Geuss, 2009) if participants are given a convincing cover story for why they are holding the rod.

We suggest that our results differ from those reported by Linkenauger et al. (2011) because on every trial in their experiment, participants were explicitly encouraged to consider their ability to grasp an object immediately before they estimated the size of that object. In contrast, participants in Experiments 2 through 4 here always estimated object size first, and in a context where attention was not drawn to action capacity, whereas in Experiment 5 participants were given a cover story to explain why we were assessing both their ability to grasp an object and their estimate of its size. Note that despite being told that the grasping and the size estimation tasks were separate and independent, the predictions of the action-specific account still hold in Experiment 5. First, participants still performed the relevant grasping action, second, they were explicitly and repeatedly told that we were interested in their grasping behavior so their attention was drawn to grasping, and third, they knew that they would have to act on each object immediately after estimating its size so they intended to act on it when they made their estimate.

In Linkenauger et al. (2011), participants were asked on each trial if they could grasp a given block so they imagined grasping the presented blocks, as opposed to actually grasping them as was done in the present studies. We did not directly test for a difference between actual and imagined grasping, and it is possible—though, we feel, unlikely—that this is a critical methodological difference. It is important to emphasize that, on every trial in Experiments 2 through 5 here, our participants always actually grasped the object by either feeling objects behind a curtain in the HV (haptic-to-vision) tasks or picking up and moved them in the VV (vision-to-vision) tasks. Therefore, what was removed from our tasks was only drawing participants’ attention to action for no apparent reason. We did not remove the action itself. We did not replicate Linkenauger et al. (2011) by testing imagined action without actual action because we do not believe that this situation occurs often in everyday life. If the action-specific account applies only when we consciously think about action, then its relevance for everyday life is severely limited. Furthermore, action-specific scaling effects have been found in previous work when participants actually acted rather than imagined doing so (e.g., Witt & Dorsch, 2009; Witt et al., 2005).

It is not entirely clear why effects consistent with the action-specific account were found in these previous experiments but not in our current work. One possibility is that spatial estimates in previous studies reflected participants’ attribution of their poor performance (in the case of Witt & Dorsch, 2009) or difficulty of the task (in the case of Witt et al., 2005) to the nature of the external stimulus, rather than to their own action capacity. This has been demonstrated experimentally. For example, although Wesp, Cichello, Gracia, and Davis (2004) reported that more successful dart throwers estimated targets as bigger than less successful throwers, in a later study Wesp and Gasper (2012) found that when participants were told that the darts were of poor quality, the association between success and estimated target size disappeared. In the original experiment, less successful throwers may have assumed the targets were smaller than they appeared and, because of this, they were harder to hit (Cole & Balcetis, 2011; Wesp & Gasper, 2012). In contrast, in the follow-up study participants could attribute their lack of success to the poor quality darts, so there was no need for them to assume the targets were smaller than they appeared, and so their estimates did not change.

This account is subtly—but importantly—different from the explanation that the action specific account would provide, namely that the targets actually looked smaller to poorer dart throwers. This alternative account instead proposes that poorer throwers may have estimated the targets as smaller to reduce the cognitive dissonance between their expectation about how good they would be at the task and the reality of their poor performance. This explanation would not apply in our studies because our participants could explore and estimate the size of all the stimuli. Even the largest blocks could be felt by moving the hand from one side to the other so participants could always estimate block size, regardless of graspability. Our results therefore suggest that performing a task-relevant action is not sufficient for action-specific scaling effects to occur.

The present studies are not without limitations. For example, we did not include a condition including conflation of estimates of perceived action capacity and spatial properties to test whether this would allow us to replicate the original Linkenauger et al. (2011) finding that perceived grasping capacity influences perceived object size. We also did not directly test whether only imagining acting would give rise to the expected action-specific effect where actually grasping did not. We therefore do not have direct evidence to support our proposal that drawing attention to the relationship between grasping capacity and spatial perception (by asking about both on every trial) caused the results of Linkenauger et al. (2011). A future study comparing their methodology with the methods used here, which were intended to dissociate action capacity and spatial perception, would be fruitful.

The results of the present experiments indicate that changes in action capacity do not affect perceived spatial properties in the strongest sense. We have suggested that there are at least two alternative explanations for previous reports of action-specific scaling effects. First, participants’ spatial estimates may have changed because of a discrepancy between their expectations about how well they would perform a task and their actual performance. Second, spatial judgments may have been conflated with perceived action capacity. In conclusion, though the relationship between perceived action capacity and spatial perception is not yet fully understood, we have demonstrated that estimates of both can be dissociated, and we found no evidence that perception is cognitively penetrable. Our results suggest that action capacity and spatial properties can be perceived independently.

## Figures and Tables

**Table 1 tbl1:** Posterior Probabilities for the Null [p_BIC_(H_0_|D)] and Alternative [(p_BIC_(H_1_|D)] Hypotheses for the Main Effects and Interactions in Experiment 2

Effect	p_BIC_(H_0_|D)	p_BIC_(H_1_|D)	η_p_^2^
Grasping Hand	.826**	.174	.01
Task	.999***	.001	.64
Prime Group	.723**	.277	.05
Grasping Hand × Task	.058	.942**	.26
Grasping Hand × Prime Group	.643*	.357	.07
Task × Prime Group	.833**	.167	.01
Grasping Hand × Task × Prime Group	.837**	.163	.004
* weak evidence. ** positive evidence. *** strong evidence.			

**Table 2 tbl2:** Posterior Probabilities for the Null [p_BIC_(H_0_|D)] and Alternative [(p_BIC_(H_1_|D)] Hypotheses for the Main Effects and Interaction in Experiment 3

Effect	p_BIC_(H_0_|D)	p_BIC_(H_1_|D)	η_p_^2^
Grasping Hand	.694*	.306	.06
Prime Group	.816**	.184	.01
Grasping Hand × Prime Group	.838**	.162	.004
* weak evidence. ** positive evidence.			

**Table 3 tbl3:** Posterior Probabilities for the Null [p_BIC_(H_0_|D)] and Alternative [(p_BIC_(H_1_|D)] Hypotheses for the Main Effects and Interactions in Experiment 4

Effect	p_BIC_(H_0_|D)	p_BIC_(H_1_|D)	η_p_^2^
Grasping Hand	.812**	.179	.01
Task	.006	.994***	.37
Tape Group	.611*	.389	.08
Grasping Hand × Task	.838**	.162	.004
Grasping Hand × Tape Group	.754**	.246	.04
Task × Tape Group	.610*	.390	.08
Grasping Hand × Task × Tape Group	.825**	.175	.01
* weak evidence. ** positive evidence. *** strong evidence.			

**Table 4 tbl4:** Posterior Probabilities for the Null [p_BIC_(H_0_|D)] and Alternative [(p_BIC_(H_1_|D)] Hypotheses for the Main Effects and Interaction in Experiment 5

Effect	p_BIC_(H_0_|D)	p_BIC_(H_1_|D)	η_p_^2^
Grasping Hand	.961***	.039	.02
Tape Group	.775**	.225	.03
Grasping Hand × Tape Group	.967***	.033	.01
** positive evidence. *** strong evidence.			

**Table 5 tbl5:** Mean (and Standard Deviation) of the Percentage of Trials Performed Using the Specified Grasp Without First Having to be Reminded by the Experimenter, for Perceived and for Actually Graspable Blocks

Hand	Before size estimation task (first grasp)		After size estimation task (second grasp)	
	Perceived as graspable	Actually graspable	Perceived as graspable	Actually graspable
Left	90% (6.6%)	93% (3.7%)	87% (7.9%)	91% (5.8%)
Right	88% (6.8%)	92% (4.4%)	86% (7.7%)	91% (4.9%)
Taped	86% (6.1%)	92% (3.5%)	83% (8.2%)	90% (5.5%)

## Footnotes

1 When included in the same ANOVA, where hand (hand/right) and estimate type (perceived/actual) were within-participants factors and prime group (LHFeelsSmallerObjects/RHFeelsSmallerObjects) was a between-participants factor, there was a marginally significant Grasping Hand × Estimate Type interaction, *F*(1, 28) = 3.88, *p* = .059, η_p_^2^ = .12.

2 When included in the same ANOVA, where Grasping Hand (left/right) and estimate type (perceived/actual) were within-participants factors and Tape Group (LHTaped/RHTaped) was a between-participants factor, there was a significant Grasping Hand × Estimate Type × Tape Group interaction, *F*(1, 28) = 45.80, *p* < .001, η_p_^2^ = .62. The nature of the Grasping Hand × Tape Group interaction was the same for both estimates types (see Figure 6), but the differences were greater for perceived than for actual grasping capacity.

3 When included in the same ANOVA, where Grasping Hand (left/right/taped) and estimate type (perceived/actual) were within-participants factors and Tape Group (LHTaped/RHTaped) was a between-participants factor, there was no three-way interaction *F*(2, 60) = 0.016, *p* = .9, η_p_^2^ = .001.

## A Results of the Perceived Object Size Tasks in Experiment 2 for All Blocks (Full Dataset)

Ratios were calculated for the visually estimated size of each block by dividing the estimated size by the actual size. These were averaged over all 21 block sizes. For the LHFeelsSmallerObjects group, one-sample *t* tests showed that ratios for the HV-left (0.76), HV-right (0.77), VV-left (0.87) and VV-right (0.86) conditions were all significantly lower than 1, *t*(14) = −8.99, *t*(14) = −7.12, *t*(14) = −4.39, and *t*(14) = −4.31, respectively, all *p* < .001. Similarly, for the RHFeelsSmallerObjects group, ratios for the HV-left (0.81), HV-right (0.82), VV-left (0.92) and VV-right (0.90) conditions were all significantly lower than 1, *t*(14) = −7.32, *t*(14) = −7.57, *t*(14) = −4.32, and *t*(14) = −4.87, respectively, all *p* < .001.

A mixed ANOVA with Grasping Hand (left/right) and Task (HV/VV) as within-participants factors and Prime Group (LHFeelsSmallerObjects/RHFeelsSmallerObjects) as a between-participants factor was conducted (*p* values for all pairwise comparisons were Bonferroni corrected). This revealed that ratios for the HV Task (0.79) were significantly lower than ratios for the VV Task (0.89), *F*(1, 28) = 40.70, *p* < .001, η_p_^2^ = .59. We found no difference between ratios for the left hand (0.84) and right hand (0.84), *F*(1, 28) = 0.07, *p* = .8, η_p_^2^ = .002, and no effect of Prime Group, *F*(1, 28) = 2.07, *p* = .2, η_p_^2^ = .07, of Task × Prime Group, *F*(1, 28) = 0.01, *p* = .9, η_p_^2^ < .001., of Grasping Hand × Prime Group, *F*(1, 28) = 0.81, *p* = .4, η_p_^2^ = .03, or of Grasping Hand × Task × Prime Group, *F*(1, 28) = 0.01, *p* = .9, η_p_^2^ < .001. However, there was a significant Task × Grasping Hand interaction, *F*(1, 28) = 4.81, *p* = .037, η_p_^2^ = .18. Pairwise comparisons showed that ratios were marginally smaller for the right hand than the left hand in the VV Task (mean difference = −0.011, *p* = .06), but there was no difference between the right and left hands in the HV Task (mean difference = −0.009, *p* = .2).

## B Results of the Perceived Object Size Task in Experiment 3 for All Blocks (Full Dataset)

Ratios were calculated for the visually estimated size of each block by dividing the estimated size by the actual size. These were averaged over all 21 block sizes. Ratios for both the left hand (0.86) and right hand (0.84) were significantly lower than 1 in the LHFeelsSmallerObjects group, *t*(14) = −4.75, and *t*(14) = −5.40, respectively, both *p* < .001. Ratios for both the left hand (0.88) and the right hand (0.87) were significantly lower than 1 in the RHFeelsSmallerObjects group, *t*(14) = −6.24, and *t*(14) = −5.93, respectively, both *p* < .001.

We conducted a mixed ANOVA, with Grasping Hand (left/right) as a within-participants factor and Prime Group (LHFeelsSmallerObjects/RHFeelsSmallerObjects) as a within-participants factor. We found no significant effect of hand, *F*(1, 28) = 2.17, *p* = .2, η_p_^2^ = .07, Prime Group, *F*(1, 28) = 0.32, *p* = .5, η_p_^2^ = .01, or a Hand × Prime Group interaction, *F*(1, 28) = 1.53, *p* = .2, η_p_^2^ = .05.

## C Results of the Perceived Object Size Tasks in Experiment 3 for Only the Blocks That Were Actually Graspable, Based on Participants’ Actual Maximum Grasp

Ratios were calculated for the visually estimated size of each block by dividing the estimated size by the actual size. For stimuli that were actually graspable in Experiment 3, ratios for the left hand (0.85) and the right hand (0.84) were significantly lower than 1 in the LHFeelsSmallerObjects group (*t*(14) = −4.76, and *t*(14) = −5.49, respectively, both *p* < .001). Ratios for the left hand (0.87) and the right hand (0.87) were also significantly lower than 1 in the RHFeelsSmallerObjects group (*t*(14) = −5.57, and *t*(14) = −6.29, respectively, both *p* < .001).

We conducted a mixed ANOVA, where Grasping Hand (left/right) was a within-participants factor and Prime Group (LHFeelsSmallerObjects/RHFeelsSmallerObjects) was a between-participants factor. There was no significant effects of Grasping Hand, *F*(1, 28) = 3.24, *p* = .08, η_p_^2^ = .10, Prime Group, *F*(1, 28) = 0.52, *p* = .5, η_p_^2^ = .02, or Grasping Hand × Prime Group, *F*(1, 28) = 0.64, *p* = .4, η_p_^2^ = .02.

## D Results of the Perceived Object Size Tasks in Experiment 4 for All Blocks (Full Dataset)

Ratios were calculated for the visually estimated size of each block by dividing the estimated size by the actual size. These were averaged over all 21 block sizes. For the LHTaped group, one-sample *t* tests showed that ratios for the HV-left (0.85), HV-right (0.85), VV-left (0.93) and VV-right (0.94) conditions were all significantly lower than 1 (*t*(14) = −4.65, *t*(14) = −4.83, *t*(14) = −2.15, and *t*(14) = −1.59, respectively, all *p* < .001). Similarly, for the RHTaped group, ratios for the HV-left (0.84), HV-right (0.83), VV-left (0.90) and VV-right (0.89) conditions were all significantly lower than 1, *t*(14) = −3.19, *p* = .007, *t*(14 = −4.75, *t*(14) = −4.29, and *t*(14) = −4.03, respectively, all *p* < .001).

A mixed ANOVA with Grasping Hand (left/right) and Task (HV/VV) as within-participants factors and Tape Group (LHTaped/RHTaped) as a between-participants factor was conducted. This revealed that ratios for the HV Task (0.84) were significantly lower than ratios for the VV Task (0.92), *F*(1, 28) = 10.37, *p* = .003, η_p_^2^ = .27. There was no significant difference between ratios for the left (0.88) and right (0.88) Grasping Hands, *F*(1, 28) = 0.09, *p* = .8, η_p_^2^ = .003, and no effect of Prime Group, *F*(1, 28) = 0.67, *p* = .4, η_p_^2^ = .02, Task × Prime Group, *F*(1, 28) = 0.24, *p* = .6, η_p_^2^ = .01, Grasping Hand × Tape Group, *F*(1, 28) = 0.39, *p* = .6, η_p_^2^ = .01, Grasping Hand × Task, *F*(1, 28) = 0.16, *p* = .7, η_p_^2^ = .01 or Grasping Hand × Task × Tape Group, *F*(1, 28) = 0.04, *p* = .8, η_p_^2^ = .002.

## E Results of the Perceived Object Size Tasks in Experiment 4 for Only the Blocks That Were Actually Graspable, Based on Participants’ Actual Maximum Grasp

Ratios were calculated for the visually estimated size of each actually graspable block by dividing the estimated size by the actual size. For the LHTaped group, one-sample *t* tests showed that ratios for the HV-left (0.88) and HV-right (0.86) were significantly lower than 1, *t*(14) = −2.56 and *t*(14) = −3.43, respectively, both *p* < .001). However, ratios for the VV-left (1.02) and VV-right (1.03) conditions were not significantly different from 1, *t*(14) = −0.39, *p* = .7 and *t*(14) = 0.43, *p* = .7, respectively. For the RHTaped group, ratios for the HV-left (0.81), HV-right (0.83), VV-left (0.89) and VV-right (0.89) conditions were all significantly lower than 1, *t*(14) = −4.15, *p* = .001, *t*(14) = −4.00, *p* = .001, *t*(14) = −3.85, *p* = .002, and *t*(14) = −3.50, *p* = .004, respectively. Marks (1978) suggested that smaller objects may appear smaller when grasped between two fingers than when seen, but this difference diminishes as object size increases. In future research we intend to investigate why estimates of object size differ for vision and touch.

A mixed ANOVA with Grasping Hand (left/right) and Task (HV/VV) as within-participants factors and Tape Group (LHTaped/RHandTaped) as a between-participants factor was conducted. Ratios for the HV Task (0.85) were significantly lower than for the VV Task (0.96), *F*(1, 28) = 16.62, *p* < .001, η_p_^2^ = .37. There was no significant effects of Grasping Hand, *F*(1, 28) = 0.03, *p* = .9, η_p_^2^ = 0.001, Tape Group, *F*(1, 28) = 2.43, *p* = .1, η_p_^2^ = .08, Task × Tape Group, *F*(1, 28) = 2.42, *p* = .1, η_p_^2^ = .001, Taped Hand × Tape Group, *F*(1, 28) = 0.42, *p* = .5, η_p_^2^ = .02, Grasping Hand × Task, *F*(1, 28) = 0.02, *p* = .9, η_p_^2^ = .01, or Grasping Hand × Task × Tape Group, *F*(1, 28) = 0.86, *p* = .4, η_p_^2^ = .001.

## F Full Instructions Given to Participants in Experiment 5^4^

Hi, so I’m coming to the end of my PhD which is all about how people use their body to perform actions and how they estimate the size of objects. Anyway, I’m getting pretty stressed because I’m running out of time to get my last bits of data so this experiment is really two separate studies rolled into one—I’m just telling you this now, because otherwise it might seem a bit strange that you’ll be doing two different tasks that do not really go together.

The first thing I’m looking at is a control study for something I’ve already tested, where I’m checking how people grasp simple blocks with their right compared with their left hand. The second thing I’m after is your ability to match the size of objects to lines on a computer screen. I should really be testing the hand grasping task and the size matching task separately but it’s hard finding participants after Easter and, like I said, I need to get this data collected really soon.

OK, so here’s what I want you to do. I want you to use your right [left] hand to grasp and pick up a block that I’ll put down here in front of you and then move it to here, to this marker. You should pick it up using your thumb on one side and any other finger on the other side, like this. If it is too big to grasp like this then you can pick it up in any way you wish, but you *must* try to grasp it with your thumb and finger first before you try anything else - is that clear? Once you’ve grasped the block, I want you to move it to this marker here. I will be recording how you choose to grasp the blocks that you are unable to pick up in this way so sometimes you might have to wait a few seconds while I write something down. So that’s the first part with the grasping done.

Then, for the second task, you’ll do the size matching. So for this one you will use the up and down arrow keys to move apart these two lines on the screen so that the distance between the inner sides of the black lines matches the width of the block—so that if you held the block up against the monitor you’d see the inner edges of the two black lines right up against the right and left edges of the block - is that clear? It’s important that you keep your body midline aligned with the block, so please do not twist your body when you start the size matching task. In fact, if it’s comfortable, you can just keep your other hand on the correct keys all the time. Once you are happy you’ve got the lines in the right place, press Enter on the keyboard. Please try to be as accurate as you can. Then just *give me the block back* and I’ll set up the next trial. You should close your eyes while I put down another block. This is because we are interested in how you grasp the blocks, and if you see how I pick them up to place them on each trial, this might influence how you then choose to grasp them. So I’ll tell you when to open your eyes and then you’ll start all over again—is that clear?

After they completed the first block for either their left or right hand, they received further instructions before starting the second block for their other hand:
Right, that’s the first block finished. Remember that I said that one of the things I was looking at here was how you grasped simple blocks with your right compared with your left hand? Well we’ve finished with the trials where you pick up blocks with your right [left] hand so now I’m going to swap things around and you’re going to do the same thing again except that you’ll be picking up blocks with your left [right] hand. You’ll also keep going with the other study, the size matching study, so this second part will be very similar to the first—on each trial you’ll pick up the block with your left [right] hand then you’ll do the size matching study then hand me the block back. Is that all clear?

Finally, after they had completed both left and right hand blocks, the fingers of one of their hands was taped in the same way as in Experiment 4, and they received further instructions:
Right, well done, that’s the second block finished. Now remember that I said that one of the things I was looking at here was how you grasped simple blocks with your right compared with your left hand? Well now I’m going to tape up your right [left] hand to see how this affects your grasping behavior. You’re then going to do just what you’ve been doing so far except you will use your taped right [left] hand to pick up the blocks. You’ll also keep going with the other study, the size matching study, so again this third part will be very similar to the first two parts – you’ll pick up the block with your taped hand then you’ll do the size matching study then hand me the block back. Is that all clear?

## G Use of Specified Grasp

We recorded whether participants performed the specific grasp required (thumb on one side and any other finger on the opposing side) without having to be reminded by the experimenter, see Table 5. If a participant did not spontaneously use the specified grasp the experimenter stopped them and told them to do this so the specified grasp was attempted on 100% of trials. For consistency with the main results section, here we only show the results for graspable blocks.[Table-anchor tbl5]

## H Results of the Perceived Object Size Tasks in Experiment 5 for All Blocks (Full Dataset)

Ratios were calculated for the visually estimated size of each block by dividing the estimated size by the actual size. These were averaged over all 21 block sizes. For the LHTaped group, one-sample *t* tests showed that ratios for the left (0.83), right (0.84) and taped (0.82) hands were all significantly lower than 1, *t*(15) = −4.38, *p* = .001, *t*(15) = −4.20, *p* = .001, and *t*(15) = −5.08, *p* < .001, respectively. Similarly, for the RHTaped group, ratios for the left (0.88), right (0.87) and taped (0.85) hands were all significantly lower than 1, *t*(15) = −4.80, *t*(15) = −4.72, and *t*(15) = −4.78, all *p* < .001, respectively.

A mixed ANOVA with Grasping Hand (left/right/taped) as a within-participants factor and Tape Group (LHTaped/RHTaped) as a between-participants factor was conducted, There were no significant effects: Grasping Hand, *F*(2, 60) = 1.42, *p* = .2, η_p_^2^ = .05; Tape Group, *F*(1, 30) = 0.73, *p* = .4, η_p_^2^ = .02; interaction of Grasping Hand × Tape Group, *F*(2, 60) = 0.08, *p* = .9, η_p_^2^ = .003.

## I Results of the Perceived Object Size Tasks in Experiment 5 for Only the Blocks That Were Actually Graspable, Based on Participants’ Actual Maximum Grasp

Ratios were calculated for the visually estimated size of each actually graspable block by dividing the estimated size by the actual size. For the LHTaped group, one-sample *t* tests showed that ratios for the left (0.85), right (0.85) and taped (0.84) hands were all significantly lower than 1, *t*(15) = −3.82, *p* = .002, *t*(15) = −3.80, *p* = .002, and *t*(15) = −4.45, *p* < .001, respectively. Similarly, for the RHTaped group, ratios for the left (0.90), right (0.91) and taped (0.88) hands were all significantly lower than 1, *t*(15) = −2.30, *p* = .036, *t*(15) = −2.15, *p* = .048 and *t*(15) = −2.29, *p* = .037, respectively.

A mixed ANOVA with Grasping Hand (left/right/taped) as a within-participants factor and Tape Group (LHTaped/RHTaped) as a between-participants factor was conducted. There were no significant effects: Grasping Hand, *F*(2, 60) = 0.24, *p* = .8, η_p_^2^ = .008; Tape Group, *F*(1, 30) = 0.95, *p* = .3, η_p_^2^ = .03; interaction of Grasping Hand × Tape Group, *F*(2, 60) = 0.06, *p* = .9, η_p_^2^ = .002.

## J Postexperimental Questionnaires for Experiments 2, 3, and 4

Prior to giving a formal debrief, in Experiments 2 through 4 all participants were asked the following three questions:

1 What did you think the experiment was testing?
2 Did you notice anything particular about the first phase of the experiment, where I gave you practice with the stimuli? (In Experiment 4, we changed this question to “What did you think the purpose of the taping was?”)
3 Do you have any ideas about why the instructions for the visual matching task were so specific?

They were then asked whether they had any further comments to make about the experiment. These three questions were scored out of 5 by the first author, where a score of 0 reflected very little to no insight into the aims of the experiment, and 5 reflected complete awareness. If the participant made any spontaneous comments about the experiment, these were also recorded.

For Experiment 2, the mean score (of 5) for Q1 was 0.5 (range = 0–4). The most commonly suggested experimental aims were that we were comparing size perception in vision and touch, and that there might be differences between the left and right hands as a result of laterality and hemispheric differences in the brain. One participant noted that they were aware that they felt that their right hand was larger than their left hand, but they did not spontaneously suggest that this may have influenced their perception of the size of objects. The mean score for Q2 was 0.1 (range = 0–1). Only three participants commented on the practice (priming) phase. They suggested that it seemed unnecessary or lengthy. The mean score for Q3 was 0 and none of our participants commented on why the instructions in the vision-to-vision (VV) task were very specific.

For Experiment 3, the mean score (of 5) for Q1 was 0.3 (range = 0–2). The most commonly suggested experimental aims were again the comparison between size perception in vision and touch, and that estimates for the left and right hands might be different because of hemispheric differences in the brain. The mean score for Q2 was 0.1 (range = 0–4). One participant noticed that larger objects were always being presented to their right hand during the practice (priming) phase but they did not suggest that this might have influenced their perceived grasping capacity. The mean score for Q3 was 0.03 (range = 0–1). One participant suggested that the instructions in the vision-to-vision (VV) task were specific because of the shape of the visual field and another suggested that the blocks might look different depending on the side of the mouse it was placed but they did not specify why. No participants correctly identified that we were trying to ensure that the same hand they had acted with remain visible for the whole trial.

For Experiment 4, the mean score (of 5) for Q1 was 0.4 (range = 0–3). The most commonly suggested experimental aims were comparing size estimation in vision and touch, the influence of handedness and effects of laterality in the brain. The mean score for Q2 was 0.3 (range = 0–4). Three participants suggested that the taping might influence their perception of object size, however they did not spontaneously predict the direction of the effect. After prompting from the experimenter one of these participants predicted that taping might lead to objects appearing bigger, another one proposed the opposite and the third did not suggest a direction even after prompting. The mean score for Q3 was 0 and no participants offered a reason for why the instructions in the VV task were very specific.

## K Postexperimental Questionnaires for Experiment 5

Prior to giving a formal debrief, in Experiment 5 all participants were asked the following five questions:

1 What did you think the experiment was testing?
2 Did you notice anything particular about the instructions?
3 Why do you think I asked you to do two separate tasks at the same time?
4 Did you think that the two tasks were related?
5 Why do you think I taped your fingers together in the last part of the experiment?

The mean score (of 5) for Q1 was 0.6 (range = 0–4). Participants rarely suggested an answer to this question, but one person suggested that the right hand would probably be better at grasping objects and so might be more accurate in the size estimation task. The mean score for Q2 was 0. Some participants commented that the specified grasp was strange, and that it was odd that they had to close their eyes. The mean score for Q3 was 0.4 (range = 0–5). One participant suggested that estimates might be lower for the right hand than the left hand because right handers are more confident with their right hand (scored 5), but most participants did not provide an answer or suggested that one of the two tasks was a distractor task. The mean score for Q4 was 1 (range = 0–5). The most common response was that the tasks were likely related but participants generally struggled to explain how. Some suggested they were related simply because the same stimuli were used in both tasks. One participant (scored 5) suggested that objects would be estimated as smaller in the right hand because right handers are more confident with their right hand. The mean score for Q5 was 1.2 (range = 0–5). Participants frequently suggested that the tape was to make the grasping task harder, and some commented that this might affect their performance in the size estimation task. Specifically, they tended to suggest that graspable blocks might be estimated more accurately than ungraspable blocks, and so they may be less accurate when their hand was taped. However, they rarely elaborated on what they meant by ‘accurate’ or mentioned size. One participant said that they noticed they had a reduced maximum grasp with their hand taped but they didn’t think this would affect their size estimates. Overall, only six participants thought we were interested in differences between the left and right hands, and four of these referred only to grasping capacity and not to estimates of object size.
